# Piezoelectric inkjet printing of tyrosinase (polyphenol oxidase) enzyme on atmospheric plasma treated polyamide fabric

**DOI:** 10.1038/s41598-022-10852-2

**Published:** 2022-04-26

**Authors:** Tuser T. Biswas, Junchun Yu, Vincent A. Nierstrasz

**Affiliations:** Textile Materials Technology, Department of Textile Technology, Faculty of Textiles, Engineering and Business/The Swedish School of Textiles, University of Borås, 501 90 Borås, Sweden

**Keywords:** Enzymes, Biomaterials, Sustainability, Materials science

## Abstract

Tyrosinase enzyme was digitally printed on plasma pretreated polyamide-6,6 fabric using several sustainable technologies. Ink containing carboxymethyl cellulose was found to be the most suitable viscosity modifier for this enzyme. Before and after being deposited on the fabric surface, the printed inks retained enzyme activity of 69% and 60%, respectively, compared to activity prior printing process. A good number of the printed enzyme was found to be strongly adsorbed on the fabric surface even after several rinsing cycles due to surface activation by plasma treatment. Rinsed out fabrics retained a maximum activity of 34% resulting from the well-adsorbed enzymes. The activity of tyrosinase on printed fabrics was more stable than ink solution for at least 60 days. Effects of pH, temperature and enzyme kinetics on ink solution and printed fabrics were assessed. Tyrosinase printed synthetic fabrics can be utilized for a range of applications from biosensing and wastewater treatment to cultural heritage works.

## Introduction

Inkjet printing of enzymes on textile surfaces can facilitate resource-efficient development of advanced products both on a small and large scale^1^. Enzymes are often used in solution form posing a challenge to recovery, downstream processing and related purification. Printing enzymes on solid support can resolve such challenges, additionally, offering possibilities of economic gain through more active and stable catalysis, resistance to denaturation and improved shelf life^2,3^. Compared to film-like supports, porous textiles can provide a larger surface area for higher enzyme loading and substrate transportation. Moreover, textile fabrics can enable large-scale production and end-uses due to their inherent strength, flexibility and lightweight. The efficiency of an enzyme immobilized on fabric depends on the used method and most of the related studies used conventional methods, such as dipping, to covalently bind enzymes on flat supports using a harsh and extensive amount of chemicals^3–7^. For example, the use of benzidine, dicyclohexylcarbodiimide and glutaraldehyde as a binding agent often involves an incubation or similar step lasting more than 18 h. Thus, it is important to explore sustainable ways of achieving the same by a facile method that uses fewer chemicals and involves digitalized technologies.

Inkjet printing is a digitally controlled system that can ensure controlled, precise and contactless deposition of biomaterials on solid supports as often required for advanced end-uses e.g. drug delivery, controlled release and bio-sensing. There is immense research and industry interest in employing this sustainable technology to replace many conventional processes that use an excess of energy, water and other chemical resources. Studies involving inkjet printing of enzymes on textile support materials are on the rise as well^1^. Direct deposition of enzymes in form of an ink liquid eliminates the issue of partial protein loading on solid supports as often experienced in conventional immobilization approaches^3–7^. Additionally, inkjet printing can facilitate enzyme deposition in specified high-resolution designs for the production of functional surfaces e.g. microarray.

Tyrosinase (polyphenol oxidase) is a copper-containing enzyme that catalyzes the hydroxylation of monophenol like L-tyrosine (4-hydroxyphenylalanine) to *o*-diphenol like L-dopa (3,4-dihydroxy-L-phenylalanine). It can catalyze the oxidation of diphenol to *o*-quinone which can go through non-enzymatic polymerization to produce melanin pigments. It is abundantly found in natural sources such as plants, vegetables and seafood. This enzyme has been investigated as a green product for a variety of applications such as oxygen sensor^5^, phenol (eco-toxin) detection in food products^3^, bioremediation of industrial wastewater^7^, biochemical conjugation^8^ and treatment of Parkinson's disease^4,9^. Tyrosinase has also been explored for the restoration of amino acid-based natural textile fibers e.g. wool and silk. The enzyme was used to crosslink peptides derived from hydrolyzed wool^10^ and enable covalent grafting of chitosan on silk^11^ to improve strength and crease-resistant properties. In general, fibre strength and crease-resistant properties were improved after enzyme treatment. Tyrosinase showed the potential to bind another protein or enzyme on an amino-acid based textile surface^12^.

Polyamide fabrics are recyclable synthetic textiles with increasing demand for applications ranging from home furnishing to medical devices^13^. Such fabric has excellent physical and mechanical properties, however inherently has low surface energy to adhere with enzyme or ink solution during a printing process. Nevertheless, the surface of PA can be activated through gas-based plasma treatment. Atmospheric non-thermal plasma is a dry, environment friendly and rapid processing technology which can replace chemical primer^4,5,14,15^ based on hazardous modifications of PA surface. The plasma technique has been used to modify PA topography, increase surface energy and introduction of functional groups^16–18^. In turn, this can promote better adsorption of enzyme protein molecules through their amino end groups^19^. Tyrosinase has been immobilized on PA in several studies^4,5,14,15^, however majority use the covalent attachment method. It has been stated to impart satisfactory immobilization, however, often using the aforementioned chemically extensive and lengthy procedures. Compared to such a method, the potential of physical adsorption based approach has not been well explored, though its importance has been mentioned in literature^7,20,21^. Adsorption is a simple and cheaper method with less probability of protein denaturation and diffusion limitations compared to other sophisticated methods^7,14^. A drawback of the adsorption method has been mentioned as the necessity of using a higher enzyme amount^21^, however, this can be solved by direct deposition of the enzyme through inkjet printing.

Despite the immense potential of printing tyrosinase on PA fabric, challenges remain due to several difficulties in their practical implementation and demand combination of different variables. First, ink containing enzyme needs to maintain certain rheological properties to ensure proper drop ejection^22^, at the same time, sustain a feasible ionic nature to retain protein stability and related activity. Then, tyrosinase must be able to withstand printing temperature and shear stress generated inside the printhead. The stability of tyrosinase has been mentioned as a challenging factor to maintain during operation^7–9^. PA fabric surface needs to be suitably modified for proper interaction with printed enzymes without compromising their activity. Reusability and prolonged storage of printed materials should be satisfactory considering practical application scenarios. This work aimed to address these challenges by combining several sustainable technologies with a holistic vision of developing products for advanced applications.

## Experimental section

### Materials

A plain weave polyamide 6,6 fabric (PA) with a weight of 118 g m^−2^ was used as support material for printing and it was kindly provided by FOV Fabrics AB (Sweden). Tyrosinase (EC 1.14.18.1) from mushroom *Agaricus bisporus* was purchased from Worthington Biochemical Corporation (USA). All other chemicals were of analytical grade and obtained from Merck/Sigma-Aldrich.

### Plasma treatment and fabric characterization

Before plasma treatment, all fabric samples were washed by using a non-ionic surfactant (1% w/w of Triton-X 100) for 30 min at 50 °C, followed by rinsing with distilled water and drying. An atmospheric pressure glow discharge equipment (PLATEX 600, Grinp, Italy) was used for plasma treatment of the fabric surface. Argon gas (1.5 L min^−1^) was used before each treatment to create an inert environment and thus facilitating homogeneous action of the reactive feed gasses. Three surface treatments were achieved by varying the feed gasses as oxygen (2 L min^−1^), nitrogen (2 L min^−1^) and a combination of these two gasses (1 L min^−1^ of each). Treatment parameters were kept constant at electrical power of 1.5 kW, feed speed of 1 m min^−1^ and inter-electrode distance of 1.5 mm.

Effects of plasma treatment of fabric surface were evaluated both qualitatively and quantitatively through scanning electron microscopy (SEM), X-ray photoelectron spectroscopy (XPS), wettability and tensile strength measurements. SEM analysis was carried out using an FEI Quanta200 ESEM (Thermo Fisher Scientific) at a low vacuum using water vapour as the gaseous environment with an accelerating voltage of 10 kV. XPS was performed on a PHI 5000 VersaProbe-III instrument equipped with a monochromated aluminium source with a photon energy of 1486.6 eV and beam size diameter of 100 µm at 15 kV. Wettability was measured through water contact angles (WCA) of the treated surfaces by using the sessile drop method on an optical tensiometer (Attension Theta, Biolin Scientific) with drop volume. WCA on three random positions was measured immediately after landing a 3 µL water drop at room temperature. Tensile strength to rupture the fabrics was measured according to ISO 13934/1 standard using a semi-automatic electronic strength tester (Tensolab, Mesdan).

### Ink formulation and printing

The ink recipe consisted of four constituents i.e. pH adjusted buffer solution, viscosity modifier, surfactant, and enzyme. Glycerol (Mw ~ 92), carboxymethyl cellulose (CMC, Mw ~ 90,000), polyethylene glycol (PEG, Mw ~ 300), polyvinyl alcohol (PVA, Mw ~ 9000) were explored as potential viscosity modifiers. The activity of the prepared inks was optimized for each modifier following the strategies of our previous work^23^. Triton-X 100 was used as a non-ionic surfactant. Inks had viscosity of 7–9 mPa s at 20 °C and shear rate 10,000 s^−1^, surface tension of 31–34 mN m^−1^ and tyrosinase protein concentration of 1 mg ml^−1^ at pH 6 (if not stated otherwise).

A drop-on-demand piezoelectric inkjet printhead (Dimatix Sapphire QS-256/80, Fujifilm, USA) with 100 dots-per-inch resolution was used for printing. It was mounted on a custom-made printing platform manufactured by Xennia technology. Inks were printed on fabric samples as a solid rectangle on an area of 6 cm × 2 cm. Each sample contained about 0.1 ml of printed ink. The printhead was set to a frequency of 35 kHz and a temperature of 30 °C (if not stated otherwise). The effect of printhead mechanism on tyrosinase activity was tested by printing on a glass plate and subsequently, collecting inks for assays. Fabric samples printed with enzyme were dried at room temperature for one hour before proceeding for activity assays.

### Activity assay

Tyrosinase activity of ink and printed fabrics were measured against 1 mM L-tyrosine substrate in phosphate buffer (50 mM) solution at 30 °C and pH 6 (if not stated otherwise). In a modified cuvette system, 0.1 ml of ink or a printed fabric was added to 2.9 ml of substrate solution and the reaction was followed by a UV–visible spectrophotometer at 280 nm. One active unit was defined as the amount of enzyme causing an increase in absorbance of 0.001 per minute. The activity was calculated from the initial linear rate against a standard calibration curve covering a range of protein concentrations (see “Results”).

### Determination of kinetic constants

Michaelis–Menten kinetic constant (*K*_*m*_) and maximum rate of the reaction (*V*_*max*_) for tyrosinase in ink and printed samples were measured from initial reactions rates against L-tyrosine with concentrations between 0.5 and 2 mM in phosphate buffer (50 mM) at 30 °C and pH 6. *K*_*m*_ and *V*_*max*_ values were calculated from Lineweaver–Burk plots.

### Protein quantification

The number of proteins released from printed fabric to phosphate buffer (50 mM) solution was counted by bicinchoninic acid (BCA) assay. Briefly, a working solution was made by adding 50 parts of reagent A (sodium carbonate, sodium bicarbonate, bicinchoninic acid and sodium tartrate in 0.1 M sodium hydroxide) and 1 part of reagent B (cupric sulfate). Then, 0.1 ml of buffer containing proteins were added to 2 ml of working solution and incubated for 30 min at 37 °C before cooling to room temperature. The concentration of proteins was measured spectroscopically by the corresponding absorbance at 562 nm against a constructed standard curve.

### Statistical analysis

The OriginLab program was used for data and statistical analysis. All presented quantitative data points are the mean of at least three observations and percent relative standard deviation was used for relative activity (%) data sets. Results mentioned as ‘significantly different’ (*p* < 0.05) were obtained by the one-way analysis of variance and the Tukey test among the two groups.

## Results and discussions

### Effect of plasma treatment

Plasma treatment can modify surface topography, increase surface energy and introduce functional groups on PA fibre^24^. and thus promote better adhesion and accessibility of biological molecules from ink and substrate solution towards fibre. Effects of such modification are dependent on several parameters of plasma treatment, such as type and amount of gas used, electric power used to energize the gasses, distance between electrodes and fabric feed speed. Among these parameters, the used gas is the main factor determining which functional group would be introduced on the PA surface. Oxygen and nitrogen are common reactive gasses used for this purpose^25^. Hence, the effects of these two gasses on PA fabric to influence adhesion and activity of printed tyrosinase were studied by keeping other plasma parameters constant. Treated fabrics were characterized qualitatively by SEM and quantitatively by XPS and WCA analyses.

**Figure 1:**
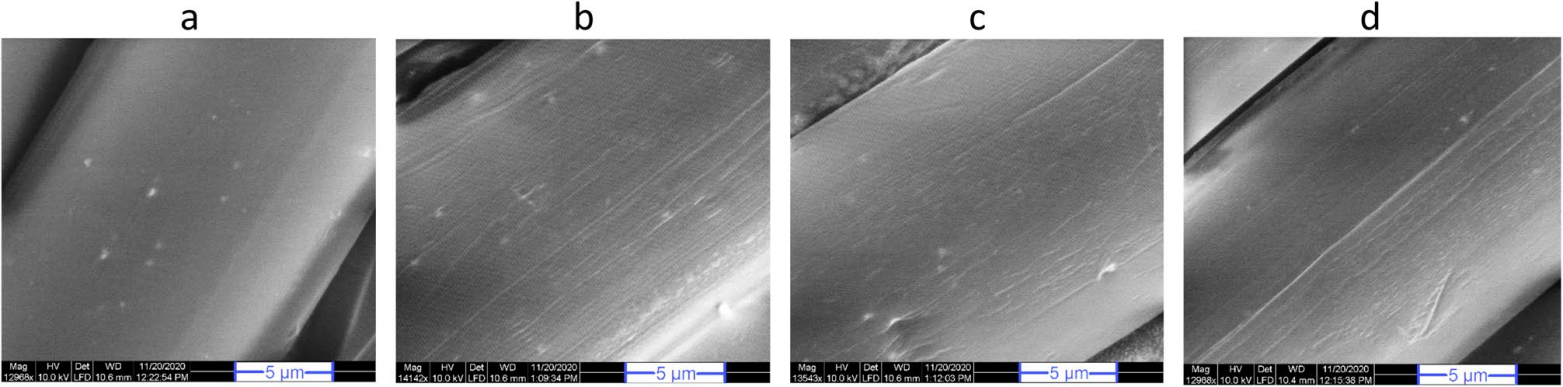
SEM images of polyamide fabrics; (**a**) untreated and plasma treated with (**b**) oxygen, (**c**) nitrogen and (**d**) oxygen + nitrogen.

SEM images (Fig. 1) confirmed the change of morphology and increased roughness of PA surface, as expected after plasma treatment^25^. Compared to the smoother surface on untreated samples, micro-etched areas were visible on all treated samples due to the bombardment of gaseous ions by the plasma process. However, it was not possible to conclusively differentiate such effects brought about by various gasses from SEM figures. Nevertheless, the achieved effects indicated improvement of fabric wettability for better ink absorption and increased surface area for higher sites available for enzyme adsorption.

XPS was used to identify molecular elements (Table 1) and functional groups present on the PA surface. As expected, C, O, and N were found as major constituents of untreated and plasma-treated fabric. For all plasma treated samples, atomic% of C reduced and atomic% of both O and N increased. Oxygen and combined gas treated fabrics had significantly higher O% (~ 5.5%) and nitrogen gas treated had higher N% (~ 4.5%). The ratio of O/C and N/C values reflected a similar trend. In term of binding energy, peaks for C–C chain and amido nitrogen [–C–NH(C=O)–] was observed at 284.8 eV and 287.6 eV, respectively, on untreated samples (see Supplementary Fig. S1). The peak intensity of amido carbonyls (C=O) was increased for oxygen plasma (~ 6.5%) at 286.6 eV and nitrogen-containing plasmas (~ 12%) at 288 eV. Such an increase confirmed successful oxidation on the PA surface caused by plasma. Further, a newly formed C–OH functional group appeared on nitrogen-containing plasma samples with 14–17.5% higher peak intensity at 285.9 eV. Similar results were found in the previous works^16–18,26^ and ensured the formation of carboxylic species in hydrocarbon or carbonyl groups of plasma-treated PA fabrics of this study.

**Table 1:** Surface elements of variously pretreated polyamide-6,6 fabrics.

	Untreated	Plasma treated with		
		Oxygen	Nitrogen	Oxygen + nitrogen
C%	75.7	70.5	69.7	68.9
O%	15.4	20.8	17.8	21.4
N%	7.8	8.5	12.3	9.6
O/C ratio	0.203	0.295	0.255	0.311
N/C ratio	0.103	0.121	0.177	0.139

WCA of all plasma treated fabrics was reduced by about 30° compared to untreated fabric (Table 2) due to an increase in surface energy and the introduction of functional groups ^17,18^. Atmospheric plasma is well known for causing chain scission of weak surface bonds on polymer surfaces leading to the creation of polar groups. This is achieved through surface oxidation during plasma treatment. The formation of new functional groups on PA surface was confirmed by XPS as stated above. However, no significant difference in WCA was found between treatments with different gasses. To enhance the effects of each applied gas, other parameters of treatment (e.g. amount of feed gas, electric power) need to be optimized. Nominal reduction of tensile strength values (1–2%) was observed after plasma treatment as this process is known to be less detrimental to the bulk properties of the fibre^24,27,28^.

**Table 2:** Water contact angle of variously pretreated polyamide fabrics.

	Untreated	Plasma-treated with		
		Oxygen	Nitrogen	Oxygen + nitrogen
Water contact angle (°)	84 ± 5	50 ± 4	51 ± 4	59 ± 3

### Ink formulation

Formulating an ink containing enzyme needs careful optimization of ionic and rheological properties, along with printhead parameters as demonstrated in our previous work^23^. Ionic profiles help an enzyme to maintain an active state, viscosity and surface tension ensure proper drop formation and ink spreading on fabric and suitable printhead adjustment helps ink flow for drop ejection. Among other ink constituents, viscosity modifiers take a large part (30–50%) and can highly influence activity values compared to an enzyme in buffer solution only^23^. Common viscosity modifiers for piezoelectric inkjet printing systems include glycerol, CMC, PEG and PVA. However, the effects of these on tyrosinase activity when used as ink formulations were not well explored. Therefore, four ink combinations were evaluated in this study to understand the effects of these modifies on tyrosinase activity (Table 3).

**Table 3:** Activity of tyrosinase (1 mg ml^−1^) in buffer and ink solutions with various viscosity modifiers.

	Buffer	Glycerol	CMC	PEG	PVA
Activity (Units ml^−1^)	958 ± 38	103 ± 16	654 ± 20	178 ± 35	745 ± 36

Tyrosinase activity of inks made with PVA (78%) and CMC (68%) showed markedly higher values than inks containing PEG (19%) and glycerol (11%) when compared to activity in the buffer. In general, the addition of large polymers in an ink solution would increase viscosity and limit molecular redistribution ability. This often causes macromolecular overcrowding, thus leading to a limited diffusion rate and reduced enzyme activity^29^. CMC and PVA can provide certain stability and protection to tyrosinase against such reduction. CMC has been found to ensure the structural stability of tyrosinase to heat and storage^3^. PVA has been described to create a hydrophobic layer around the enzyme and thus, protection against invasive polymerization^8^. Any such protection has not been observed to offer by glycerol and PEG^8,30^.

**Figure 2:**
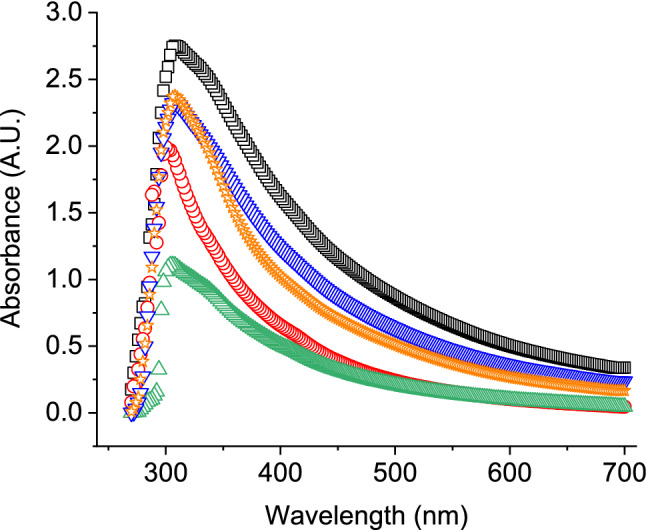
UV–visible absorption spectra of tyrosinase in buffer (□, black) and ink solutions made with glycerol (○, red), carboxymethyl cellulose (∇, blue), polyethylene glycol (Δ, green) and polyvinyl alcohol (☆, orange) as viscosity modifiers.

UV–visible spectroscopy of tyrosinase in buffer and prepared ink solution exhibited a broad band of absorbance around 300–320 nm (Fig. 2). Tyrosinase absorbance peak around this wavelength range has been observed in previous studies due to the presence of tyrosine, phenylalanine, tryptophan and histidine residues^31–33^. However, absorbance intensity was significantly different between prepared inks. Glycerol and PEG containing ink had lower intensity and indicated unwanted interaction between enzyme and solvent^8,30^. Intensity of CMC and PVA containing inks was close to that of buffer, suggesting well preservation of protein structure residues of enzyme. Additionally, this ensured uniformity and well dispersion of ink composing materials to avoid printhead nozzle blocking.

**Figure 3:**
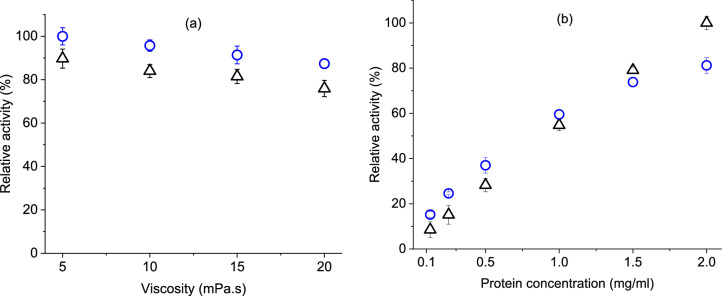
Activity of tyrosinase ink containing carboxymethyl cellulose (∆) and poly-vinyl alcohol (O) as viscosity modifiers at various (**a**) ink viscosities (same protein concentration) and (**b**) protein concentrations (same viscosity). Results are expressed as percentages against the highest activity found for each graph.

Suitable ink viscosity to print through a piezoelectric printhead can vary between 5 and 20 mPa s, depending on the system requirements^22^. An enzyme would be expected to perform efficiently over such a viscosity range. However, there was a lack of studies regarding the effect of viscosity variation on tyrosinase activity concerning CMC and PVA modifiers during printing or similar mechanical processing. Therefore, four inks were printed in this study for each of the modifiers over a range of viscosity and keeping the protein concentration constant (Fig. 3a). For both modifiers, a slight reduction of activity (maximum ~ 10%) was observed with increased viscosity. As already mentioned, this might have been caused by diffusion limitation of enzymes with increased modifier amount. Over the same viscosity value, these two groups of inks showed no significant difference in the activity. Unlike other enzymes^23^, no initial increase in activity was seen due to the addition of CMC or PVA. These results suggested that both of the modifiers could be used for printing tyrosinase over the general viscosity range of industrial printheads.

It was important to find a linear range of activity over enzyme concentration for these two modifiers. This would help to define the initial velocity and substrate saturation ability of prepared inks. Thus, two groups of inks with both modifiers were tested in this study covering a range of protein concentrations (0.1–2.0 mg ml^−1^) with a negligible change of viscosity. Results showed (Fig. 3b) that PVA containing inks were able to maintain linear activity until 1 mg ml^−1^ of enzyme protein concentration. Although, CMC containing inks maintained a linear relationship until the highest used concentration (2 mg ml^−1^). Such difference between the two modifiers might be related to their molecular weight and resulted in protein overcrowding^34^. In general, it could be concluded that CMC based inks would ensure more reasonable activity readings at comparatively higher protein concentrations which might occur on fabric after drying of printed ink.

### Printability and ink stability

Theoretical printability of both CMC and PVA based inks was evaluated through limiting values of a few dimensionless characteristics numbers i.e. Webers number (*We*) and inverse Ohnesorge number (*Z*). These numbers were calculated from density and surface tension of the ink, along with velocity and characteristics length of the printhead as explained in literature^35^. Found numbers (*Z* = 2.8–3.7, *We* = 10.4–11.6) were well within the range for efficient inkjetting process (1 < *Z* < 10; *We* > 4)^36^. These theoretical values ensured that during the printing process inks would overcome the influence of the air-fluid interface that is necessary for drop formation. Furthermore, continuous ejection through printhead nozzles would take place without the formation of satellite drops.

Initially, CMC and PVA based inks were printed and collected on a glass plate to check the effects printhead actuation mechanism on tyrosinase activity. CMC based ink retained higher activity (69%) than PVA based ink (52%) when compared to respective activity before printing. As observed for piezoelectric printing of other proteins^28,37^, such reduction of activity could be caused by shear stress generated inside the printhead during drop ejection to influence tyrosinase protein structure. Activity difference between CMC and PVA based inks might be related to the solvent composition which can interfere with the electrostatic forces governing protein conformation^38^. CMC has been suggested to provide a hydrophilic microenvironment to minimize such modification and ensure better protection to tyrosine active structure^34,39^. Additionally, PVA based ink was more challenging to print with a lower amount collected on a glass plate and requiring frequent purging of the printhead. Therefore, CMC based ink was selected to further investigate and printed on plasma-treated fabrics.

**Figure 4:**
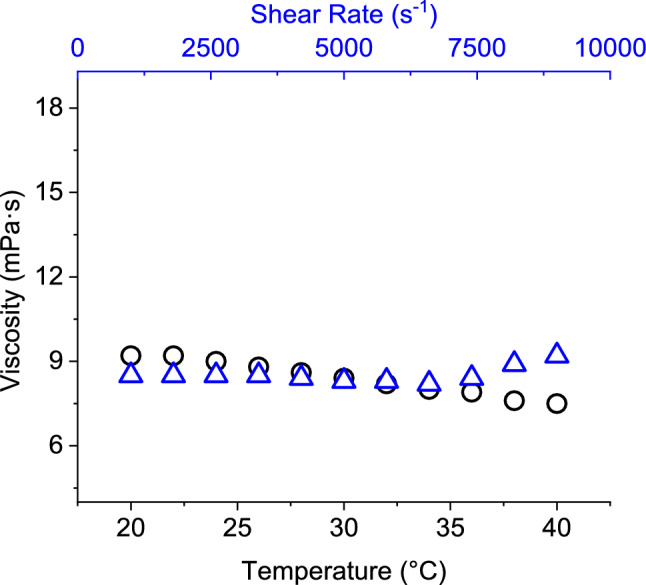
Effect of temperature (O) and shear rate (∆) on the viscosity of carboxymethyl cellulose based ink.

The stability of ink should be ensured during the printing operation and storage period. Stability could be affected by variation of ionic profiles, rheology, temperature and interaction between ink constituents. As presented in Fig. 4, prepared CMC based ink showed significantly stable viscosity of 7.5–9.2 mPa·s and 8.2–9.2 mPa·s over a range of probable printhead temperatures (20–40 °C) and shear rate (100–10,000 s^−1^), respectively. An ink with Newtonian behavior is expected to be suitable for most of the recent piezoelectric printheads^22^. Local variation of viscosity due to inkjetting force and heat development inside the printhead would be less probable for such an ink. Therefore, even fluid flow and efficient drop ejection were likely to result from the prepared CMC based ink.

**Figure 5:**
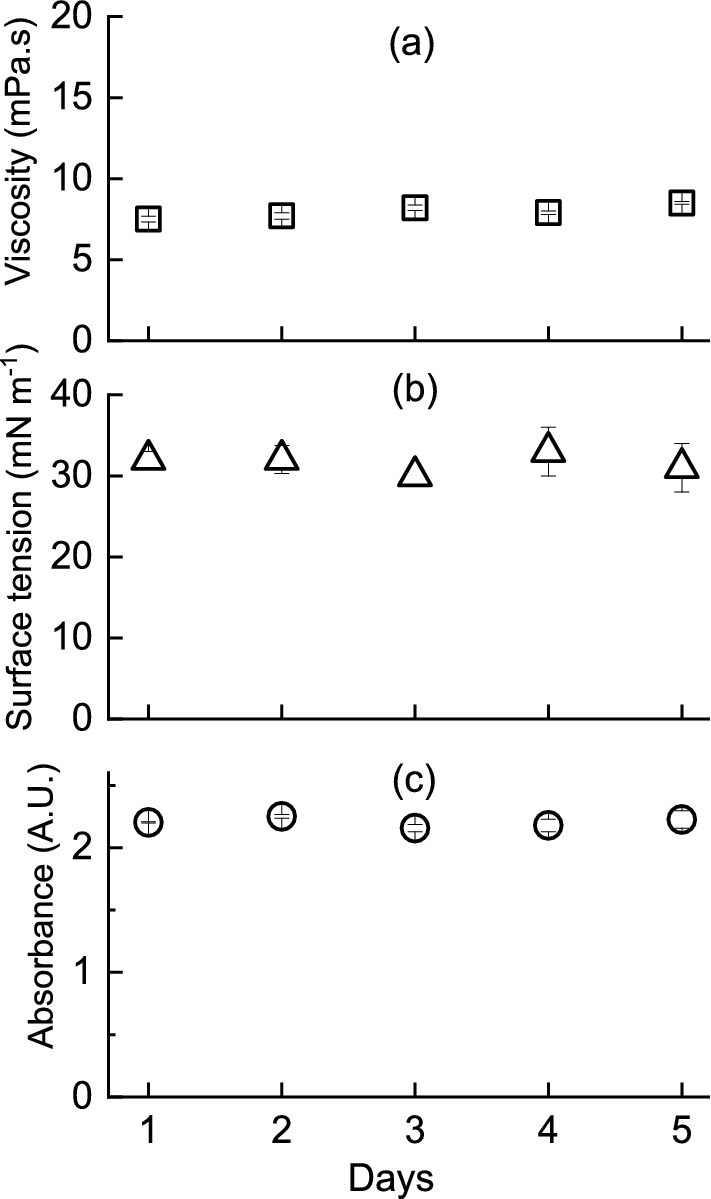
Stability of ink (**a**) viscosity (□), (**b**) surface tension (∆) and (**c**) absorbance at 280 nm (Ο) over time.

The prepared ink was stored for 5 days at 4 °C and stability to several variables was tested (Fig. 5). UV–visible absorbance at 320 nm ranged 2.16–2.25 (arbitrary unit) over the storage period, indicating good compatibility among ink materials and less likelihood of unwanted interference. Surface tension was maintained between 30 and 33 mN m^−1^, confirming well dispersion of ink particles and avoidance of adsorption on the surface of the ink bottle, tubing or printhead inner surfaces. Rapid change of viscosity that could indicate particle aggregation was unlikely as it was stable around 7.5–8.5 mPa s. Additionally, the pH value of ink during storage was stable and ensured proper maintenance of enzyme protein structure.

### Tyrosinase activity

**Figure 6:**
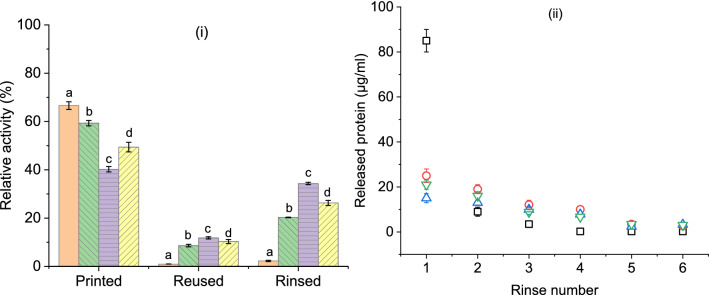
(**i**) Activity of tyrosinase printed on polyamide-6,6 fabrics (a) untreated and plasma-treated by (b) oxygen, (c) nitrogen and (d) mixture of oxygen and nitrogen gasses, remaining activity of the same fabrics upon one reuse and activity after six rinsing cycles in buffer solution. Results are presented as a percentage of the ink activity before printing (100% activity); (**ii**) Protein (tyrosinase) released in buffer solution upon several rinsing cycles from fabrics: untreated (□) and plasma pretreated by oxygen (O), nitrogen (Δ) and the mixture of oxygen and nitrogen (∇) gasses.

Activity found on printed fabrics was significantly lower (33–60%) than the activity of ink solution (Fig. 6i). Such a reduction might have been resulted by a change of enzyme–substrate interaction from a macro (ink) to a microenvironment (fabric) and corresponding issues of diffusion. Additionally, activity reduction could be influenced by various immobilization phenomenon subjected to the enzyme e.g. change of protein structure and interaction with fiber matrix^3,4^. After an activity assay, fabric samples were removed from the cuvette and subjected to a second assay cuvette to check reusability. A maximum of only 12% activity (nitrogen plasma treated) was observed among all samples during reuse. Further, freshly printed fabrics were dried and rinsed in buffer solution for several cycles to release any loosely adsorbed enzyme proteins. After the sixth rinsing cycle (Fig. 6ii), protein release became negligible and then the samples were subjected to an activity assay. Surprisingly, fully rinsed fabrics showed higher activity (max. 34%) than reused ones. Few studies found similar or even higher activity after immobilization and reuse of tyrosinase^6,7,14,15^. However, most of those studies used uncontrolled and lengthy dipping or incubation based methods of enzyme deposition, contrary to the controlled and rapid approach of inkjet printing. Hence, it is difficult to compare the findings of this study with the literature, nonetheless, it can be regarded as satisfactory for pioneering inkjet printing of tyrosinase on plasma-treated PA fabric.

Found tyrosinase activity among printed fabrics was dependent on received pretreatment (Fig. 6i). Untreated fabrics retained higher activity after printing but were lost almost entirely upon the reuse and rinsing process. Plasma treated fabrics retained similarly low activity (9–12%) upon reuse irrespective of the used gasses. After the rinsing process, nitrogen gas treated fabrics showed significantly higher activity (34%) than the other two treatments (20–26%). Though, before the rinsing process, nitrogen gas treated fabrics had lower activity (40%) than the other two groups (49–59%). Such activity variations were regulated by the nature and stability of enzyme adsorption on fabric surface which was further reflected through the release of printed enzymes upon rinsing (Fig. 6ii). After the first rinsing cycle, protein release was significantly lower for plasma treated fabrics (15–25 µg ml^−1^) than untreated ones (~85 µg ml^−1^). This meant that most enzymes from untreated fabrics were reacting at a homogeneous state with substrate solution and showed higher activity than plasma treated fabrics. It explained low activity values upon reuse as many proteins were already released. Additionally, product contamination from the first cuvette might have influenced the reuse assay. Although the released enzymes were in the same state of ink during catalysis, their activity could not reach the same level due to the possible compromise of protein structure that occurred during adsorption-desorption processes^40^.

Enzymes released after the fourth rinse cycle became negligible and enzymes that remained on fabric surface after the sixth rinse cycle could be considered as strongly adsorbed. Lower activity from this group of fabrics could simply attribute to less amount of available enzymes. Additionally, these enzymes were reacting solely in a heterogeneous state and could show reduced activity due to a changed orientation of active sites towards fiber rather than substrates^40^. Plasma treated fabrics could facilitate better adsorption of enzymes due to increased surface roughness and specific area, alongside, probable hydrophilic and ionic interaction^41^. SEM images showed the creation of micro-etched areas (Fig. 1) and XPS results (Table 1) confirmed the increment of oxygen and nitrogen species after respective plasma processes, along with the introduction of hydroxyl and carboxyl groups. Nitrogen gas containing treatments showed relatively higher activity which might have been aided by better adsorption kinetics between plasma-enhanced amide groups on fabric^25^ and amino groups on enzyme protein molecules^42^.

### Effect of pH and temperature

**Figure 7:**
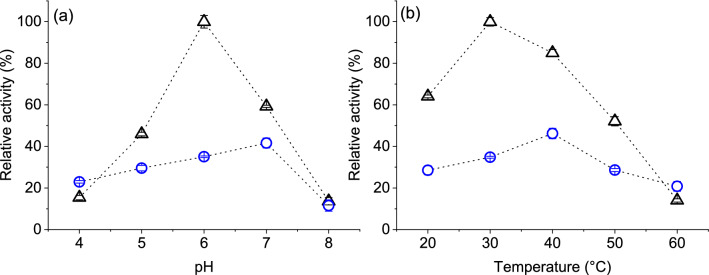
Effect of (**a**) pH and (**b**) temperature on the activity of tyrosinase containing ink (∆) and printed-rinsed polyamide fabrics (O). Results are expressed as percentages against the highest activity found for each graph.

The effectiveness of tyrosinase printed fabric could be demonstrated by safeguarding an optimum pH and temperature. Enzyme adsorption on a solid surface, substrate affinity and activity are often regulated by surrounding pH and temperature. The value of pH can influence the net surface charge of these amphoteric protein molecules and active conformation. Higher temperature increases the reaction rate, however until a limit before starting to bring irreversible conformational changes i.e. denaturation. Therefore, the effect of these two variables was studied on nitrogen plasma treated PA fabric printed with tyrosinase and after rinsing the same for six cycles. Fabric samples had significantly low activity than ink samples for most pH and temperature ranges (Fig. 7). This might have resulted simply due to reduced protein amount on fabrics after the rinsing process as already discussed.

Ink solution had optimum activity around pH 6 (Fig. 7a) and significantly lower activity at other pH values as expected for tyrosinase^43^. This result indicated that the response to solution ionic properties of the enzyme was not altered by ink formulation constituents. The activity of printed enzymes that remained on fabric was optimum around pH 7 (~ 42%) and the change of activity among pH values was less drastic compared to ink solution. A shift of optimum pH level towards the neutral region has been reported in the literature^3,9,44^ and this could indicate immobilization of enzymes on fabric surface through strong adsorption. Such a shift could be caused by the partitioning of hydrogen ions due to a charged microenvironment at the fabric surface and resulting in localized pH^45^. Measured values by a pH meter would represent the pH of assay solution, however, the activity of printed enzymes was influenced by pH near protein active sites immobilized in fabric. Negative ions induced by nitrogen-based plasma on fabric could cause an apparent shift i.e. pH 6 near printed enzymes that were read as pH 7 from assay solution. Indeed, it demonstrated that printed enzymes could be protected at higher pH compared to ink solution^3,44^, along with less activity variation over several pH values for fabric samples.

The highest activity of tyrosinase in ink form was found at an assay temperature of 30 °C and then reduced significantly with increased temperature, reaching as low as 14% at 60 °C (Fig. 7b). Comparatively, the variation of activity for printed enzymes was less drastic from the near room (~ 29%) to the respective optimum temperature of 40 °C (~ 46%). At the highest assay temperature, printed enzymes held 21% activity, which was better than ink activity under same condition. Similar to the outcomes from pH-variation experiments, a shift of optimum temperature could indicate immobilization of printed enzymes and related changes in chemical and physical properties brought about by fabric microenvironment as observed in previous studies^3,9,46^. Immobilization of an enzyme on a fibrous matrix can reduce its conformational mobility and preserve tertiary structure even at elevated temperatures^47^. The structure of enzymes in ink form probably started to destabilize around 40 °C, however, remained relatively stable in fabric. Thus, inkjet-printed tyrosinase would be less susceptible to pH and temperature conditions compared to its use in free solution form.

### Kinetic studies

The kinetic constants (*V*_*max*_ and *K*_*m*_) were calculated over a range of substrate concentrations for tyrosinase in ink solution and printed on nitrogen plasma treated PA fabric (rinsed). Both of the constants were reduced significantly for fabric samples (Table 4). In general, kinetic parameters change upon immobilization on porous fabric like support due to diffusional limitation of substrates, steric hindrance towards the active site and lack of protein conformation flexibility^40^. *V*_*max*_ was expected to reduce due to such immobilization effect, in addition to the fact that there was less amount of enzyme available on fabric after the rinsing process. A reduced *K*_*m*_ meant higher affinity between enzyme and substrate and thus, another reason for lower *V*_*max*_ resulted from fabric samples. *K*_*m*_ could be apparently reduced due to partitioning of charged molecules in the fabric microenvironment. Under the kinetics assay conditions, the concentration of a positively changed substrate at low ionic strength (50 mM) could appear to be higher near active site of the printed enzyme than the same on bulk form i.e. ink solution. Therefore, a lower substrate concentration would be sufficient to half-saturate printed enzymes compared to enzymes present in ink solution and thus, apparently reducing the kinetic constants^45^. Similar results have been reported by other studies on tyrosinase immobilization^14,44,46^, however none related to printing technology.

**Table 4:** Kinetic parameters of tyrosinase in free-form (ink) and after printing-rinsing process (fabric).

Enzyme in	*V*_*max*_ (Units mg^−1^ protein)	*K*_*m*_ (mM)
Ink solution	1546 ± 13	1.444 ± 0.093
Fabric	408 ± 12	0.808 ± 0.001

### Storage stability

**Figure 8:**
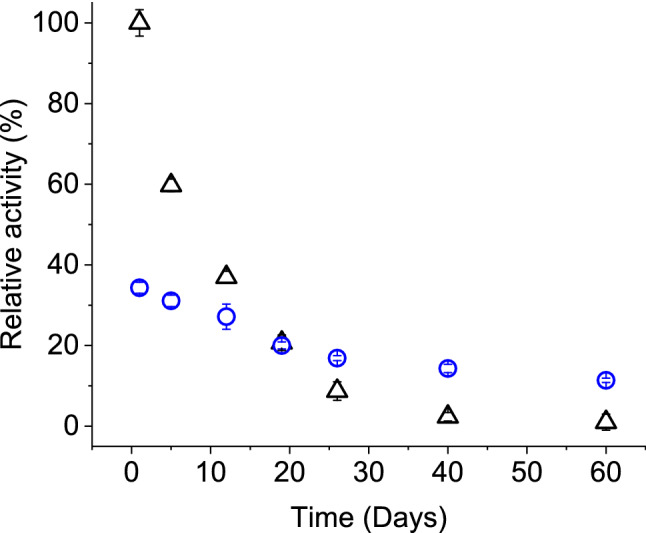
Change of tyrosinase ink (∆) and printed fabric (O) activity during storage period.

Enzymes may undergo irreversible changes in protein structure depending on storage condition and duration. This would lead to compromised activity and challenges for practical applications. Generally, immobilizing enzymes in fabric like solid support could enhance the activity period^2^. Stability of tyrosinase activity in ink solution and printed on nitrogen plasma treated PA fabric (rinsed) were evaluated during storage at 4 °C for 60 days (Fig. 8). All samples showed a gradual reduction of activity, however to different extents. The initial activity of ink samples was reduced to about half within 5 days of storage, while it took 26 days for fabric samples to reach the same level. After 40 days, ink samples lost almost all activity, whereas fabrics retained 41% of initial activity. At the end of 60 days storage period, fabrics still retained almost one-third of activity. A less drastic reduction for fabric samples could be attributed to adsorption mediated stabilization of tyrosinase protein structure compared to its free form in ink solution^48^. Other studies on tyrosinase immobilization have reported widely varied yield and duration of storage stability depending on the applied method and nature of attachment^3,4,14,34^. In general comparison, the results of this study could be further improved to attain a similar level of stability.

## Conclusion

This work showed the possibilities of combining several resource-efficient technologies for inkjet printing of tyrosinase on polyamide fabric with the potential for many advanced applications. Several ink formulations containing tyrosinase were evaluated for the maintenance of enzyme activity and stability upon printing. Carboxymethyl cellulose as a viscosity modifier retained satisfactory activity over a range of viscosity and protein concentrations and showed overall better ink performance than other modifiers. This ink maintained stable viscosity, surface tension and absorbance value over a minimum period of 5 days which are essential for successful inkjet printing. Polyamide-6,6 fabrics were plasma treated with several gasses to facilitate attachment of enzymes through strong adsorption as opposed to conventional chemical-intensive processes. Fabrics that were plasma treated with nitrogen gas sustained a higher amount of adsorbed enzyme and activity compared to the other gas combinations. This fabric retained a good number of enzymes even after several rinsing cycles. Optimum pH-temperature profile for the activity of printed fabrics shifted to a slightly higher value than for the enzyme in ink solution. Tyrosinase on fabric samples maintained stable activity over several pH and temperature ranges than in ink form. During 60 days of the storage period, initial activity from printed and rinsed fabrics was lower than ink solution. However, fabrics maintained higher activity from day twenty and thus more suitable for longer usage.

## Supplementary Information

Supplementary Information.

## Data Availability

All data generated or analysed during this study are included in this published article.

## References

[1] Biswas TT, Yu J, Nierstrasz VA, Cavaco-Paulo A, Nierstrasz VA, Wang Q (2019). Chapter 12. Advances in Textile Biotechnology.

[2] Chaplin MF, Bucke C (1990). The Economic Argument for Immobilization.

[3] Yakup Arıca M (2000). Immobilization of polyphenol oxidase on carboxymethylcellulose hydrogel beads: Preparation and characterization. Polym. Int..

[4] Pialis P, Jimenez Hamann MC, Saville BA (1996). L-DOPA production from tyrosinase immobilized on nylon 6,6. Biotechnol. Bioeng..

[5] Tungel, R., Rinken, T., Rinken, A. & Tenno, T. Immobilisation and kinetic study of tyrosinase for biosensor construction (1999).

[6] Firooz NS, Panahi R, Mokhtarani B, Yazdani F (2017). Direct introduction of amine groups into cellulosic paper for covalent immobilization of tyrosinase: Support characterization and enzyme properties. Cellulose.

[7] Abdollahi K, Yazdani F, Panahi R (2017). Covalent immobilization of tyrosinase onto cyanuric chloride crosslinked amine-functionalized superparamagnetic nanoparticles: Synthesis and characterization of the recyclable nanobiocatalyst. Int. J. Biol. Macromol..

[8] Jahangiri E (2012). Medium engineering to enhance mushroom tyrosinase stability. Biochem. Eng. J..

[9] Munjal N, Sawhney SK (2002). Stability and properties of mushroom tyrosinase entrapped in alginate, polyacrylamide and gelatin gels. Enzyme Microb. Technol..

[10] Lantto R (2005). Enzymatic modification of wool with tyrosinase and peroxidase. J. Text. Inst..

[11] Freddi G (2006). Tyrosinase-catalyzed modification of Bombyx mori silk fibroin: Grafting of chitosan under heterogeneous reaction conditions. J Biotechnol.

[12] Fu J (2015). Enzymatic processing of protein-based fibers. Appl. Microbiol. Biotechnol..

[13] Vadicherla T, Saravanan D, Muthu SS (2014). Roadmap to Sustainable Textiles and Clothing: Eco-friendly Raw Materials, Technologies, and Processing Methods.

[14] Donato L, Algieri C, Rizzi A, Giorno L (2014). Kinetic study of tyrosinase immobilized on polymeric membrane. J. Membr. Sci..

[15] Harir M (2018). Isolation and characterization of a novel tyrosinase produced by Sahara soil actinobacteria and immobilization on nylon nanofiber membranes. J. Biotechnol..

[16] Chen F (2016). Atmospheric pressure plasma functionalized polymer mesh: An environmentally friendly and efficient tool for oil/water separation. ACS Sustain. Chem. Eng..

[17] Pappas D (2006). Surface modification of polyamide fibers and films using atmospheric plasmas. Surf. Coat. Technol..

[18] Xiang C, Etrick NR, Frey MW, Norris EJ, Coats JR (2020). Structure and properties of polyamide fabrics with insect-repellent functionality by electrospinning and oxygen plasma-treated surface coating. Polymers.

[19] Morent R, De Geyter N, Cools P (2015). Advances in Bioengineering.

[20] Hu J, Mok YK, Saddler JN (2018). Can we reduce the cellulase enzyme loading required to achieve efficient lignocellulose deconstruction by only using the initially absorbed enzymes?. ACS Sustain. Chem. Eng..

[21] Bonnet C, Andreescu S, Marty J-L (2003). Adsorption: An easy and efficient immobilisation of acetylcholinesterase on screen-printed electrodes. Anal. Chim. Acta.

[22] Magdassi S, Magdassi S (2010). Chapter 2. The Chemistry of Inkjet Inks.

[23] Biswas TT, Yu J, Nierstrasz VA (2019). Effects of ink characteristics and piezo-electric inkjetting parameters on lysozyme activity. Sci. Rep..

[24] Marcandalli B, Riccardi C, Shishoo R (2007). Plasma Technologies for Textiles.

[25] McCoustra MRS, Mather RR (2018). Plasma modification of textiles: Understanding the mechanisms involved. Text. Prog..

[26] Nuhiji E (2012). Biofunctionalization of 3D nylon 6,6 scaffolds using a two-step surface modification. ACS Appl. Mater. Interfaces..

[27] Reglero Ruiz JA, Trigo-López M, García FC, García JM (2017). Functional aromatic polyamides. Polymers.

[28] Biswas T, Yu J, Nierstrasz V (2021). Effective pretreatment routes of polyethylene terephthalate fabric for digital inkjet printing of enzyme. Adv. Mater. Interfaces.

[29] Derham BK, Harding JJ (2006). The effect of the presence of globular proteins and elongated polymers on enzyme activity. Biochim. Biophys. Acta.

[30] Ikehata K, Nicell JA (2000). Characterization of tyrosinase for the treatment of aqueous phenols. Bioresour. Technol..

[31] Duckworth HW, Coleman JE (1970). Physicochemical and kinetic properties of mushroom tyrosinase. J. Biol. Chem..

[32] Shang C (2018). The effect of 7, 8, 4′-trihydroxyflavone on tyrosinase activity and conformation: Spectroscopy and docking studies. Luminescence.

[33] Shen Z, Wang Y, Guo Z, Tan T, Zhang Y (2019). Novel tyrosinase inhibitory peptide with free radical scavenging ability. J. Enzyme Inhib. Med. Chem..

[34] Borisova B (2015). A layer-by-layer biosensing architecture based on polyamidoamine dendrimer and carboxymethylcellulose-modified graphene oxide. Electroanalysis.

[35] Saunders RE, Derby B (2014). Inkjet printing biomaterials for tissue engineering: Bioprinting. Int. Mater. Rev..

[36] Liu Y, Derby B (2019). Experimental study of the parameters for stable drop-on-demand inkjet performance. Phys. Fluids.

[37] Ashton L, Dusting J, Imomoh E, Balabani S, Blanch EW (2009). Shear-induced unfolding of lysozyme monitored in situ. Biophys. J..

[38] Haghbeen K, Saboury AA, Karbassi F (2004). Substrate share in the suicide inactivation of mushroom tyrosinase. Biochim. Biophys. Acta (BBA) – Gener. Subj..

[39] Araque E (2014). Water-soluble reduced graphene oxide-carboxymethylcellulose hybrid nanomaterial for electrochemical biosensor design. ChemPlusChem.

[40] Chaplin MF, Bucke C (1990). Kinetics of Immobilised Enzymes.

[41] Kerkeni A, Behary N, Dhulster P, Chihib N-E, Perwuelz A (2013). Study on the effect of plasma treatment of woven polyester fabrics with respect to nisin adsorption and antibacterial activity. J. Appl. Polym. Sci..

[42] Liu Z, Deng J, Li D (2000). A new tyrosinase biosensor based on tailoring the porosity of Al2O3 sol–gel to co-immobilize tyrosinase and the mediator. Anal. Chim. Acta.

[43] worthington-biochem. *Tyrosinase assay manual*, http://www.worthington-biochem.com/TY/default.html (2021).

[44] Kıralp S, Toppare L, Yağcı Y (2003). Immobilization of polyphenol oxidase in conducting copolymers and determination of phenolic compounds in wines with enzyme electrodes. Int. J. Biol. Macromol..

[45] Chaplin MF, Bucke C (1990). Effect of Solute Partition on the Kinetics of Immobilised Enzymes.

[46] Çil M, Böyükbayram AE, Kıralp S, Toppare L, Yağcı Y (2007). Various applications of immobilized glucose oxidase and polyphenol oxidase in a conducting polymer matrix. Int. J. Biol. Macromol..

[47] Chaplin MF, Bucke C (1990). Effect of Temperature and Pressure: Fundamentals of Enzyme Kinetics.

[48] Canofeni S, Di Sario S, Mela J, Pilloton R (1994). Comparison of immobilisation procedures for development of an electrochemical PPO-based biosensor for on line monitoring of a depuration process. Anal. Lett..

