# Supplementary material for: Piezoelectric inkjet printing of tyrosinase (polyphenol oxidase) enzyme on atmospheric plasma treated polyamide fabric
Source: Sci Rep. 2022 Apr 26;12:6828. doi: 10.1038/s41598-022-10852-2 (PMC9043184; doi:10.1038/s41598-022-10852-2)
Supplement: Supplementary file 1 — Supplementary Information. [file 41598_2022_10852_MOESM1_ESM.docx]

Piezoelectric inkjet printing of tyrosinase (polyphenol oxidase) enzyme on atmospheric plasma treated polyamide fabric

Tuser T. Biswas*, Junchun Yu, and Vincent A. Nierstrasz

Textile Materials Technology, Department of Textile Technology, Faculty of Textiles, Engineering and Business / The Swedish School of Textiles, University of Borås, 501 90 Borås, Sweden

E-mail: [tuser.biswas@hb.se](mailto:tuser.biswas@hb.se)


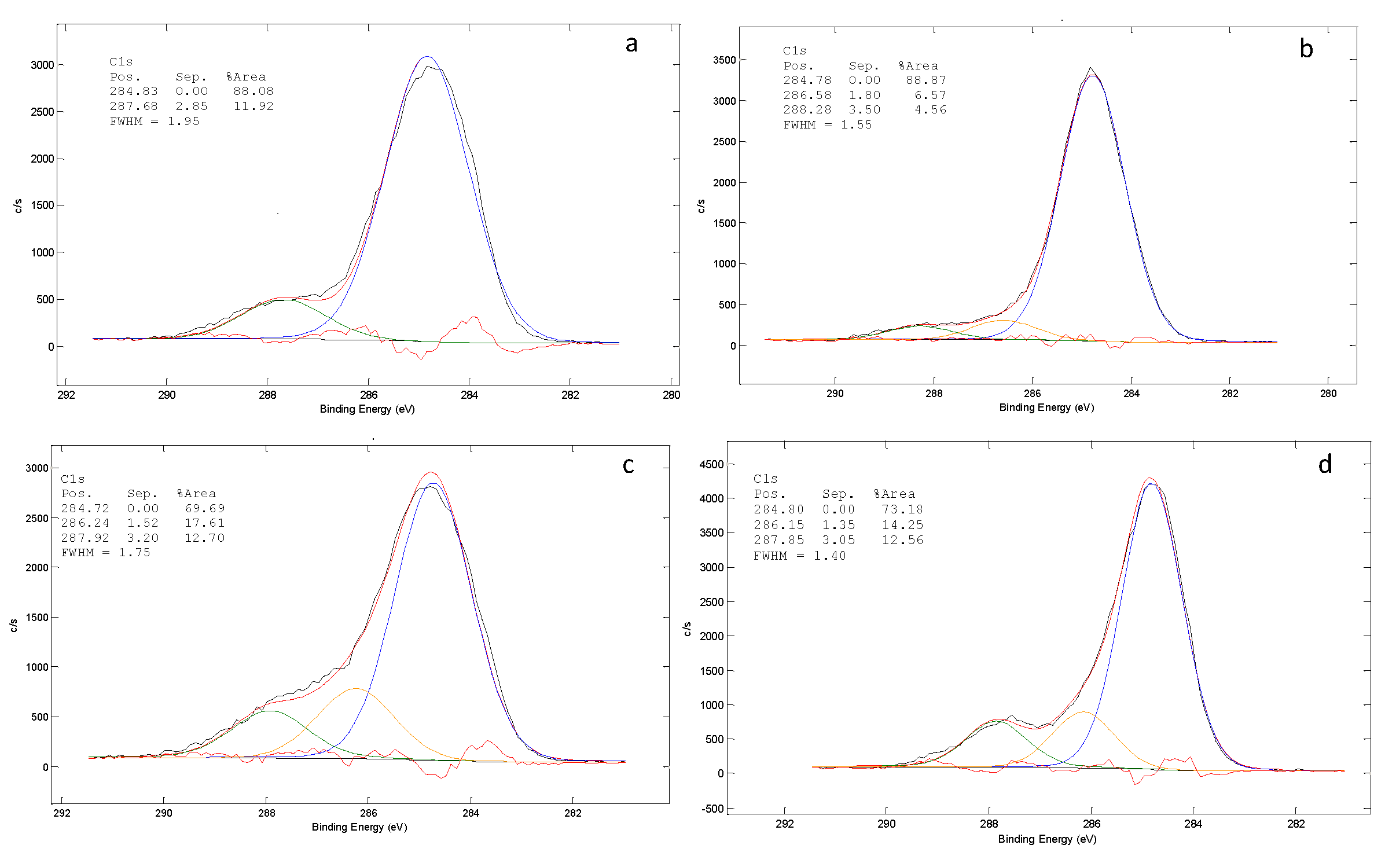


Supplementary Figure 1. XPS C1s spectra of polyamide-6,6 fabrics; untreated (a) and plasma treated with oxygen (b), nitrogen (c), oxygen + nitrogen (d)
